# Local Sanitary Conditions and Epidemics

**Published:** 1880-05

**Authors:** 


### Notes and Extracts.

### LOCAL SANITARY CONDITIONS AND EPIDEMICS.

In the number of this journal for March we copied an article from Supplement No. 3, *National Board of Health Bulletin*, giving results of investigations concerning the sanitary or unsanitary condition of the water supply of many places in the South, where yellow fever had prevailed in the summers of 1878–9. Regarding everything that can add to our knowledge of the local sanitary conditions as of paramount importance in the study of etiology, and following up the same general purpose, we shall copy below somewhat lengthy extracts from a report, in the same Supplement, on the "Sanitary History of Memphis, being based upon a house-to-house inspection of the city, between November 24, 1879, and January 3, 1880, made under the direction of the National Board of Health."

This inspection, made by parties carefully selected for their fitness to do the work, almost immediately after the prevalence of a very severe epidemic of yellow fever two summers in succession, may be regarded as fairly representing the condition of the soil, water, drainage, sources of pollution, etc., that actually existed during the two preceding seasons. And by comparing these with the meteorological conditions during the same period, as recorded for the Signal Service Bureau of the general government, we are enabled to study successfully the bearing of the entire local sanitary conditions, not only on the original progress of the epidemics, but also on the ratio of sickness and mortality from the prevalence of ordinary diseases. The latter, though attracting less public attention, is really more important to the permanent welfare of the people than the former. Severe epidemics rarely affect communities, except at long intervals, and only for a brief period of time; while a high ratio of mortality, from ordinary diseases, shows a constant sacrifice of life that, in the aggregate, far exceeds that produced by the most severe epidemics. This is fully demonstrated by the facts embodied in the table of mortality for five consecutive years, given below.

That table shows an annual prevalence of dysentery, diarrhœa, cholera infantum, pneumonia, phthisis, etc., equal to the most densely populated and unsanitary cities, either in this country or Europe; the annual death rate for the non-epidemic years being 35 per 1,000 of the population. And more than fifteen per cent. of this extraordinary ratio of mortality was from pneumonia and phthisis. But we will let the facts, as given in the report, speak for themselves, by copying as follows:—[ED.

The city of Memphis, at present the "taxing district of Shelby County," is situated in Shelby County, Tennessee, latitude 35° 7′ north, longitude 90° 7′ west, with a greatest altitude of 287.44 feet above tide-water in the Gulf of Mexico.

### POPULATION AND GROWTH.

At this time [1852] the population was estimated at less than 10,000, a rapid advance being claimed for the two subsequent years, so that in 1854 a census showed 12,867 inhabitants, which was increased by the close of 1857 to an estimated population of 25,-000, this rapid growth being attributed to the completion of the first portion of the Memphis and Charleston railroad. Although there was a large increase between 1850 and 1860, this latter estimate was not borne out by the figures of the eighth United States census, which gave Memphis a population in 1860 of 22,621, as against 8,841 in 1850, by the same authorty. The ninth census (1870) placed the population at 40,226; but it is important to note, in this connection, that the city limits were materially enlarged in 1857–'68, so much so that they embraced 4,114 acres at the time when the ninth census was taken; and much of this additional area was as thickly settled as the average of the former or present area. During the session of the State legislature of 1871 and 1872 another change was made, and the area was reduced to the present city limits, a reduction which cut off 37 + per cent. of the territory and 39 + per cent. of population. This would make the population of 1870, contained within the area of the present city limits, 24,253, and it is this figure, and not 40,226, which should be taken in any estimate of the growth of the city within the past decade.

PRESENT POPULATION.

On the 1st of January, 1880, the population of Memphis, as shown by the house-to-house inspection returns, is 30,659, showing an increase of nearly 26.4 per cent. since 1870. Of these there are 16,705 whites in 3,775 families, an average of 4.42 persons in each family, and 13,954 colored in 3,609 families, an average of 3.87 persons to each family—a mean average of 4.15 persons to the aggregate of 7,384 families. These occupy 5,584 of the 9,386 dwellings accounted for, giving an average of 5.49 persons to each occupied dwelling. In 1870 there were 5.14 persons to each family (white and colored not separated), and 6.28 persons to each dwelling. The reduced average of occupants to dwellings is readily enough accounted for by the large number of buildings erected during the decade—an increase equal to nearly 54 per cent., while the increase of population is, as above shown, less than 27 per cent. An explanation of the reduction in the number of persons to a family is more difficult, and is complicated with questions involving a study of the epidemics of 1873, 1878, and 1879, of the changed social status of the colored people, and other factors, not, probably, beyond the scope of a "sanitary survey," but for which no opportunity has yet offered.

AREA AND TOPOGRAPHY.

An area of 2,590 acres is embraced within the present city limits, the outlines of which approximate the figure of a truncated right-angled triangle (or trapezoid), the truncated extremity (8,175 feet) being the northern boundary; the perpendicular (13,050 feet) the east boundary; the base (11,625 feet) the southern boundary; while the hypothenuse (17,000 feet) is the water frontage on the Mississippi river.

The site of Memphis and the country immediately surrounding it is the Fourth Chickasaw Bluff, a loess formation, consisting of a fine siliceous loam, of a rich chrome yellow or buff color. This is superimposed upon a varicolored stratum of sands and gravel, locally known as the "orange sand," a southern extension of the drift formation, and immediately beneath which is found the La Grange group of clays and sands of the Tertiary period. The "orange sand" stratum is nearly horizontal, so that it is reached at varying depths according to the thickness of the loam above it.

The bluff formation is well defined along the southern half or two-thirds of the river front of the city, where it presents a bold, almost perpendicular western face, cut through by street openings to the levee. Its greatest elevation is in the southwestern section (on Tennessee street, near the corner of Talbot), where it rises to a height of 67.9 feet above the highest water mark, that of 1867. From this point, along the river front, it gradually descends toward the north, until it is lost in the valley of Bayou Gayoso, some 1,500 feet south of the north boundary. Toward the south the inclination is much less, so that at the south boundary near the river, the highest bluff level is still 57 feet above high water. North of Bayou Gayoso the bluff again rises, but recedes from the river at a much greater angle. Extending back (east) from the river the surface is undulating, with the long axes of the swells and depressions in general north and south lines, except in the northern portion ("Chelsea," "Scotland," and the upper part of "The Pinch"), where the long axes run nearly east and west.

### NATURAL DRAINAGE SYSTEM.

Through the depressions flow a series of bayous which form the natural drains of the area, only a narrow strip along the river front draining into the Mississippi river, except in the extreme northwestern portion of the city. Of these bayous the Gayoso and Quimby are the most important. Bayou Gayoso (with its east and west forks, and its tributaries, the Little Betty and De Soto) drains the southern and central divisions of the city, and flows in a general northerly direction until it it receives Bayou Quimby with the drainage of the northern division, when it pursues a northwest and west course, emptying into the Wolf river at a point about 2,500 feet above the junction of the latter stream with the Mississippi.

This natural drainage, flowing mainly from south to north, is still further supplemented by minor undulations running at nearly right angles with the above. As the original conformation of the site has not been materially changed, these undulations afford lateral drainage communicating with the main system, and, with the exception of the localities to be hereafter specified, this surface drainage is ample, even for the heaviest rainfalls.

The average width of the bayous within the city limits varies from ten to twenty-five feet, measured at about mid-height of the banks, and the volume of water varies with the rainfall and stage of water in the Mississippi.

There are but few springs emptying into the bayous, so that their contained water is mainly made up of the surface drainage, and of what is locally known as the "sipe" water—water which has penetrated below the surface, and gradually oozed out through the banks of the bayous. During high water in the Mississippi the river water enters the bayous and "backs up" the contents of the latter, this "backing up" having been known to extend for over a mile through the most densely settled parts of the city, and leaving behind it, in the bed of the lower part of the bayou, accumulations of mud or silt of five or six feet in depth. As the stage of water in the river is high enough to obstruct the bayou for four or five months in the year, the current is sufficiently retarded during that period to allow of a large deposit of organic matter upon the banks of the bayous, which rapidly decomposes and becomes offensive as the water falls and exposes it to the action of the sun and air. In addition to this source of pollution, there are a large number of privies, built directly over the bayous or upon their banks, the contents of which are discharged directly into the waters, while the surface and sipe water, for a distance of several hundred feet in many localities, is also contaminated with fecal filth from surface privies, overflowing and leaky vaults, etc. A five-foot sewer, which gathers the surface drainage of an area of some 140 acres, on which is the city hospital, and several small private sewers also contribute their quota to the fouling of Bayou Gayoso.

Including the city, an area of upward of 5,000 acres is drained by the bayou system; the total length of Bayou Gayoso, from Wolf River to its head, being five and a half miles, with an inclination of 1 foot in 180 in the city limits; the total length of Bayou Quimby, from its junction with Bayou Gayoso to its head, is a little less than four and a half miles, with an inclination of about one foot in 140; and the total length of Bayou De Soto, from its junction with Bayou Gayoso to its head, is two and four-fifths miles, with a slightly greater inclination than that of Bayou Quimby.

RIVERS—THE WOLF AND MISSISSIPPI.

Wolf river enter the city in the extreme northwestern section, constituting practically, its northwestern boundary for an extent of about 4,000 feet.* It is an exceedingly tortuous stream, flowing through cypress swamps and cane-brakes for about 65 miles, and receives the Loosa Hatchie river just at the northern boundary of the city. Like the bayous, it is "backed up" when the Mississippi rises more than 15 feet above low-water mark, and it is asserted that at such times the foul waters of the bayou also enter and pollute it. A tannery and a group of slaughter-houses, located about 300 yards below the water-works, discharge blood, offal and refuse into the stream through a small "run," upon whose banks they are built. It is also stated that when this "backing up" occurs such material is necessarily carried up to the pumps. Aside from its malarial influence, derived from the "bottom" lands through which it flows, the chief sanitary importance of the Wolf consists in its being, to a large extent, the source of the water supply of the city, as above intimated. Its waters have been analyzed by Assistant Surgeon Smart, United States Army, and detailed information concerning it will be found in his report upon the water supply of Memphis.

It should be added, however, that personal observation does not confirm the statements made as to the pollution of the water supply by the "backing up" of the bayou and river. With a

* In the northwestern portion of the city a section of low alluvial land about 300 acres in extent is separated from the first and ninth wards by the Wolf river. This is subject to overflow during high water, is partly cultivated in cotton, has one white and twelve or fourteen colored families (who are obliged to vacate during high water), and possesses no features of sanitary interest apart from those which characterize the Mississippi "bottom" generally.

stage of about 21 feet of water in the Mississippi, and while the bayou was still " backed up," a decided under current was found in the Wolf opposite the water-works. It is obvious that any rise in the Mississippi sufficient to dam up the waters of Wolf river would also dam up the contents of the bayous and of the " run " above alluded to; and that as the water falls the outward current in the Wolf will be re-established, carrying out with it the retained contents of the bayous and " run." The contents of these latter can only be carried up Wolf river to the water-works when to a suitable stage of water in the Mississippi shall be added a local rain (confined to the watershed of the bayous) heavy enough to swell the volume of water discharged by the bayou beyond the volume of water contained in the Wolf, and sufficiently greater to overcome the fall in the latter stream from the water-works to the mouth of the bayou. Remote as such a coincidence would seem to be, it is stated to have occurred twice within the past ten years. There is, however, reason for believing, from personal observation, that the surface water of the Wolf may be carried up stream for a considerable distance, even when the deeper water is flowing out. Whether this ever occurs to such an extent as to carry the foul waters of the bayou and " run " up to the pumps it is not presumed to assert.

The extreme range between high and low water in the Mississippi at Memphis is about 35 feet, and a gradually shelving shore of varying extent is thus alternately covered and exposed. At the date of inspection, Dec. 16, 1879, with a stage of 19 feet 10 inches above low-water mark, this shore had a greatest width of 258 feet from the foot of the bluff at Jefferson street. Extending north from Jefferson street to the mouth of Wolf river (2,687 feet measured from center of Jefferson to center of Jackson street, along the line of Front Row) the water covered this sloping shore, to the foot of the bluff, with the exceptions noted in the reports of a special inspection of the river front (*q. v.*). Extending south from Jefferson street the bluff has been cut away so as to increase the depth of the shore, and this space, known as " the levee," had a varying exposure of from 150 to 260 feet between the water's edge and the foot of the bluff until the line of Beale street was reached (nearly the same distance as that described above). At Beale street the water again came up to the foot of the bluff, but from this point south to the city limits there was an irregular width of from 20 to 200 feet.

THE WATER FRONT.

These three divisions of the river front present strongly marked contrasts. The northern one, beginning at Wolf river, has a low, flat area of about four acres ("Happy Hollow") partially submerged during high water, and receiving a considerable portion of the filthy surface drainage of the northern end of the "Pinch," one of the most objectionable quarters of the city, as well as the polluted discharge from the bayous. The bank is sandy and easily washed, so that mattress protection is necessary. From Market street south to Jefferson the bluffs gradually rise, varying in height from four to twenty-five feet, and along this extent there are gullies, "wash-outs," deposits of fecal filth, the outlets of box-drains and culverts, and the accumulations of a public "dump," including everything offensive, from street sweepings to the contents of privy vaults, which, up to May, 1879, were carted to the crest of the bluff at the foot of Washington street, and thence allowed to find their own way to the river. The central division consists of the "levee," so called, an improved area paved with stone, clean and in good condition, with the exception of such nuisances as arise from the want of public latrines for the use of the roustabouts, stevedores, rivermen, etc. As the river falls after high water a deposit of mud covers the "levee," but this is washed from the well-paved and steep slope by the first rain. The southern division reproduces many of the evils of the northern one, aggravated by the location of the present public "dump-boat" at the foot of Beale street, a decided nuisance at this date.

The details of the conditions above generalized are embraced in the special report previously alluded to, the substance of which was conveyed to the proper authorities through the committee on the sanitary survey on the 18th of December, 1879, supplementing previous reports on some of the conditions which had been already submitted on November 10 and December 13.

SEWERAGE.

There is practically no sewer system in Memphis, the four and a half miles of existing private sewers having only two hundred and fifteen connections in all. And while the natural facilities for surface drainage are ample, they have not only not been fully utilized or preserved, but in many instances they have been materially impaired, and in some entirely destroyed, by changes in the original conformation, or by the character of the street conservancy. With an extremely retentive soil this obstruction of natural drainage renders unpaved streets and alleys almost impassable during wet weather, and readily accounts for the large proportion of damp or wet cellars and basements. These causes —namely, the absence of sewers and of subsoil drains and the obstructed surface drainage—also affect unfavorably the areas of "made land," although these are not numerous or extensive. (Such areas are described in the summary description of the wards.)

STREETS AND ALLEYS.

There are sixty-seven miles of streets and thirty-five miles of alleys within the city limits. West of the bayou these run nearly with the cardinal points of the compass, the variation being a little east of north and south of east. In the northeastern section this variation is more decided, but in the central and southern sections the same general lines are preserved on both sides of the bayou. Details as to width, paving, and condition of streets, alleys, and sidewalks will be found in the ward summaries.

BUILDINGS—MATERIAL, AGE AND CHARACTER.

Of the 7,202 buildings of all kinds (this is exclusive of outhouses) within the city limits, 72 per cent. are of wood, 27 per cent. of brick, and the remainder of stone (13) and iron (1). In the strictly residence portion of the city—in fact, in all but the third and fourth wards—the large majority of the buildings are of one story, there being (with the exceptions above noted) 4,188 dwellings of one story, as against 2,198 of two or more stories. Many of the structures returned as "occupied dwellings" are, in effect the cabins of one or two rooms which, prior to 1861–'62, constituted the slave-servants' quarters. Of these (one or two roomed dwellings) there are 1,209 returned; and this item is of consequence in connection with the remarks which follow concerning the condemnation of buildings.

About 10 per cent. of the total number of all buildings have been erected within the past five years, and nearly one-fourth of the total number were erected between 1870 and 1875. These buildings, 2,524 in number, embrace the large majority of the really healthy habitations, and substantial, well-ventilated and properly lighted business blocks. A large number of buildings erected between 1865 and 1870 are mere shells, with thin, insecure walls, flimsy and badly fitting wood-work, and inadequate ventilation and lighting. There are few exceptions to the indictment of this class. Of the remainder (about 40 per cent. of the total number), the ages range from fifteen to fifty years, and there are few of these which do not require radical alterations in order to justify their retention for occupancy.

One of the most important defects noticeable in dwellings is the want of subventilation, 1,453 dwellings being built so close to the ground as to have no air-space beneath the floor; 2,030 others have insufficient or obstructed subventilation; and only 2,204 (about two-fifths of the total number) comply with the proper requirements in this respect.

### CELLARS AND BASEMENTS.

There are 1,515 buildings with cellars and basements, and of these over one-half (786) are badly ventilated, damp or wet, many with water standing from two to eighteen inches deep on the floors, and with walls soaked by sipage from the surrounding polluted soil. As very few of these cellars are more than 9 feet deep—fully one-half being between seven 7 and 8 feet, and about one-fourth being less than 7 feet deep—any general system of sewerage and subsoil drainage will remedy this latter defect.

In the two principal business wards of the city (the third and fourth) more than one-third of the buildings were found to have privy vaults in cellars or basements, Many of these buildings are upward or twenty years old, and the cellars contain from one to five vaults each, the accumulations of an average of a quarter of a century being imperfectly covered over with ashes or earth.

Not infrequently the rain-water cistern, from which water for all purposes is used, was found surrounded by these vaults with the walls almost touching each other. A few instances are to be found in every part of the city; but the majority are grouped where they are likely to do the most harm—in the densely built and oldest regions.

Among the minor subterranean defects found were 426 cellars and basements fouled by accumulations of decomposing organic matter, infected material, etc. (The past tense is here used advisedly, since much of this has been already remedied through the efforts of the board of health, which has caused most of these cellars to be cleansed and whitewashed and the vaults emptied, disinfected and filled up.)

### SOIL AND WATER POLLUTION.

So much has been written and said, *ad nauseam*, of the privy system of Memphis that it is proposed to dismiss it here in a very summary manner, and this the more justifiably, since there is a strong probability of its soon being numbered among the things of the past. At the date of inspection there were found nearly 6,000 sub-surface vaults in use. These varied from mere shallow pits, without any lining, to brick vaults of forty feet (and upward) in depth. Considerably less than one-third were sufficiently remote from living-rooms, while the remainder were placed at all degrees of proximity, even to being, as already stated, grouped in cellars. Of the total number, 3,607 were in a foul condition. The extent of the soil pollution from this source may be better inferred when it is understood that the above figures do not include a large (probably equal) number of disused, but unemptied, vaults, the contents of which were only imperfectly covered by a shallow layer of ashes or refuse.

When it it is considered that of the 4,744 cisterns and wells which furnish the large share of the water consumed, 3,408 are within contaminating distance of the known locations of these vaults, and that a large proportion of such cisterns and wells are known to be defective, enough will have been said on these points to indicate some of the causes of the high death-rate of Memphis.

MORTALITY IN MEMPHIS.

An analysis of the mortality records of the health office [for the past five years furnishes the following table, which is of interest in this connection:

| Causes of death. | Years. | | | | | Totals. |
|---|---|---|---|---|---|---|
| | 1875. | 1876. | 1877. | 1878. | 1879. | |
| Malarial fever | 127 | 99 | 121 | 126 | 68 | 541 |
| Typhoid and typho-malarial fever | 23 | 37 | 41 | 19 | 18 | 138 |
| Cerebro-spinal malarial fever | 16 | 14 | 17 | 18 | 1 | 66 |
| Yellow fever | 0 | 0 | 0 | 2,779 | 497 | 3,276* |
| Erysipelas | 6 | 1 | 6 | 1 | 12 | 26 |
| Dysentery | 66 | 79 | 63 | 30 | 25 | 263 |
| Diarrhœa | 30 | 67 | 61 | 46 | 36 | 240 |
| Cholera infantum | 52 | 21 | 31 | 19 | 32 | 155 |
| Scarlatina | 1 | 49 | 17 | 1 | 0 | 68 |
| Diphtheria | 5 | 8 | 13 | 11 | 1 | 38 |
| Croup | 13 | 4 | 5 | 6 | 11 | 39 |
| Whooping-cough | 25 | 7 | 1 | 20 | 1 | 54 |
| Small-pox | 8 | 0 | 0 | 0 | 0 | 8 |
| Measles | 0 | 13 | 2 | 35 | 0 | 50 |
| Pneumonia | 88 | 87 | 108 | 83 | 136 | 502 |
| Phthisis | 172 | 159 | 180 | 176 | 143 | 830 |
| All other diseases of lungs | 18 | 25 | 28 | 34 | 10 | 115 |
| Diseases of the heart | 27 | 27 | 28 | 29 | 26 | 137 |
| Diseases of the urinary organs | 2 | 6 | 11 | 1 | 5 | 25 |
| Tetanus | 10 | 11 | 13 | 7 | 5 | 46 |
| Puerperal diseases | 17 | 6 | 17 | 14 | 21 | 75 |
| All other causes | 468 | 308 | 491 | 552 | 520 | 2,339 |
| Totals | 1,174 | 1,028 | 1,454 | 4,007 | 1,568 | 9,230 |

From the foregoing it will be seen that, exclusive of the mortality from yellow fever, the average death-rate of Memphis is thirty-four per thousand, assuming the average population for the past five years to have been 35,000. On the census of 1879 the total mortality for that year was fifty-one in the thousand, and exclusive of yellow fever it was thirty in the thousand. The average death-rate from all causes during the three non-epidemic years was thirty-five. Of the total number of deaths during the past five years over fifteen per cent. were due to phthisis, pneumonia, and other diseases of the lungs, the excess being fairly attributable to defective sub-ventilation of dwellings and to an undrained retentive soil. Excremental and malarial diseases caused nearly fifty-seven per cent. of the total, and this excess may be set down as due, in great measure, to soil and water pollution.

Under a reasonably efficient sanitary *régime*, with a good sewerage system and pure water supply, the average death-rate of the city should be reduced to about twenty in the thousand within the next lustrum. Until the social and moral status of the colored population is materially improved, it will probably be too much to expect that the theoretical standard of seventeen in the thousand can be attained.*

* Without any special relevancy to the above, but simply to record the information, the following data concerning epidemic diseases in Memphis are here appended. These have been obtained from the note-books of physicians and from other sources among the citizens, and are believed to be trustworthy so far as they go.

YELLOW FEVER.—First epidemic in 1828: distributed pretty generally throughout the village, at that time occupying the bluff near the Mississippi, and mainly north of Market street; over 150 cases in a population of about 700; mortality, 56 recorded deaths. Second epidemic in 1855; confined principally to area south of Union street (South Memphis and Fort Pickering,) but followed the bayou on both sides, north and west to Wolf River; estimated number of cases 1,250 in a population of about 13,000; mortality 220 (estimated.) Third epidemic in 1867: included area of epidemic of 1855, and extended north and east; estimated number of cases, 2,500 in a population of 36,000; mortality, 500 to 550. Fourth epidemic in 1873; spread pretty generally throughout the city, but more severe in northern portion ("The Pinch," "Chelsea," and eighth ward,) and extended east beyond city limits; estimated number of cases, 7,000 out of a population reduced by flight to between 15,000 and 20,000: mortality, recorded between September 14, and November 9 (last death), 1,244; estimated total mortality from August 10 (first death) upward of 2,000. Fifth epidemic in 1878; no portion of city or suburbs exempt; number of cases 17,600 out of remaining population of 19,500: mortality, 5,150 recorded deaths. Sixth epidemic in 1879; area as general as in 1878, except in localities depopulated by flight or removal to camps; total number of recorded cases between July 9 and November 15 (cases occurred in December) 1,532 out of an estimated remaining population of 18,500, of which number 75 per cent. were "protected" in the sense of having previously had the disease: total recorded mortality, 485. The disease was brought to the city in several other years, but was not communicated to the inhabitants. This was notably the case in 1853, when upward of 80 imported cases were treated in hospital and boarding-houses without any spread.

CHOLERA.—First appearance in the winter of 1832-'33; number of cases and mortality unknown, but it is described as having been severe and general. Second epidemic in 1835; said to have been between 300 and 400 cases, with a mortality of about 15 per cent.; no data of population at this time, but probably about 2,400. Third epidemic in 1849: estimated number of cases about 1,200 in a population of less than 8,000; particularly severe among river boatmen and the foreign population in "The Pinch"; mortality said to have been about 33 per cent. Fourth epidemic in 1867; about 600 cases, mostly among negroes; mortality unknown; disease most severe along Causey street and vicinity (line of contact of fifth and sixth wards.) Fifth epidemic in 1873; about 1,000 cases, with 276 recorded deaths; entire population is said to have been afflicted with choleraic diarrhœa.

In addition to the foregoing, small-pox was epidemic in 1835 and in 1873; influenza in 1842; dysentery in 1844 (local, confined to "The Pinch," about 400 cases, and between 40 and 50 deaths;) jaundice, erysipelas, and puerperal fever in 1853; and dengue in 1860. Prior to 1850 diarrhœal diseases were excessively prevalent, attributed to use of well and spring water: claimed to have diminished on substitution of cistern water.

